# Yolk-shell structured magnetic mesoporous organosilica supported ionic liquid/Cu complex: an efficient nanocatalyst for the green synthesis of pyranopyrazoles

**DOI:** 10.3389/fchem.2023.1235415

**Published:** 2023-09-06

**Authors:** Maryam Neysi, Dawood Elhamifar

**Affiliations:** Department of Chemistry, Yasouj University, Yasouj, Iran

**Keywords:** periodic mesoporous organosilica, yolk-shell structure, ionic liquid, nanocomposite, pyranopyrazoles

## Abstract

The preparation of yolk-shell structured magnetic mesoporous composites is a significant subject between researchers. Especially, modification of theses composites with ionic liquid/metal complex is very important for catalytic processes. In the present study, a novel magnetic methylene-based periodic mesoporous organosilica (PMO)-supported ionic liquid/Cu complex with yolk-shell structure (YS-Fe_3_O_4_@PMO/IL-Cu) was prepared *via* the soft template-assisted method. The TGA, FT-IR, SEM, EDX, XRD, VSM, nitrogen-sorption, and ICP techniques were employed to identify YS-Fe_3_O_4_@PMO/IL-Cu. The YS-Fe_3_O_4_@PMO/IL-Cu material was applied as a powerful nanocatalyst for the synthesis of pyranopyrazoles under ultrasonic media. The study demonstrated that the YS-Fe_3_O_4_@PMO/IL-Cu nanocatalyst is highly recyclable, selective, and effective. The leaching test was performed to investigate the nature of the designed catalyst under the applied conditions.

## 1 Introduction

Yolk-shell structured nanoparticles (NPs) are hybrid materials in which a core is encapsulated in a hollow shell and can move freely within this shell, commonly demonstrated as core/void/shell. In this structure, the core is not blocked and thus provides an effective active site for the chemical processes (Kim et al., 2002; Kamata et al., 2003; Yin et al., 2004; Gao et al., 2007; Liu et al., 2013; Purbia and Paria, 2016; Xie et al., 2017). The unique properties of yolk-shell structured materials, such as low density, high surface area, permeable shells, high thermal stability, and interstitial hollow spaces, make them powerful platforms for biotechnology/biomedicine, controlled release, magnetic resonance imaging, data storage, catalysis, environmental remediation, etc. (Kresge et al., 1992; Vartuli et al., 1994; Holmes et al., 1998; Vartuli et al., 1998; Tsuji et al., 1999; Morishita et al., 2006; Puanngam and Unob, 2008; Du and He, 2011; Zhao et al., 2011; Ghaedi et al., 2013; Zhang, 2013; Nasab and Kiasat, 2016; Purbia and Paria, 2016). Among the various categories of yolk-shells (YSs), magnetic composites with Fe_3_O_4_ cores and nano-silica shells are very attractive due to their advantages such as good magnetic properties, high chemical and thermal stability, non-toxicity, high adsorption capacity, high surface area, high biocompatibility, and high accessibility of–OH groups on their surface for any modification (Arruebo et al., 2006; Liu et al., 2011; Yang et al., 2015). Recently, the catalytic application of YS-structured magnetic mesoporous silica nanocomposites has received much attention. Some of the newly developed systems in this regard are Au@Void@PMO (Yang et al., 2015), Fe_3_O_4_@SiO_2_@Pd/HSPMO (Dai et al., 2017), PMO-MHS (Zhang et al., 2008), and Fe_3_O_4_@void@mSiO_2_ (Qiu et al., 2015).

Periodic mesoporous organosilica (PMOs), a desirable class of organic-inorganic composite materials with great properties such as high surface area, high lipophilicity, and high thermal and mechanical stability, have emerged as an ideal support (Wang et al., 2015; Yu et al., 2019; Norouzi et al., 2020; Neysi and Elhamifar, 2023). In particular, bifunctional PMOs (BPMOs), which contain organic functionalities on both the mesoporous walls and channels, are highly attractive for catalytic processes

On the other hand, ionic liquids (ILs) have attracted tremendous attention in chemistry and materials science in the last decade owing to their unique characteristics, such as low vapor pressure, high chemical and thermal stability, and their capability to dissolve a variety of compounds. In particular, recently, imidazolium-based ILs have been widely used as linkers for the effective immobilization of catalytic active sites on solid supports (Neysi et al., 2020; Jangra et al., 2021; Veysipour et al., 2021; Neysi and Elhamifar, 2022).

The preparation of pyranopyrazole derivatives has emerged as a powerful tool in organic synthesis because they are an important class of biologically active compounds. Some biological properties of pyranopyrazoles are anticancer, antifungal, anti-anxiety, antiviral, and anti-AIDS (Babaie and Sheibani, 2011; Moosavi-Zare et al., 2013; Zolfigol et al., 2013; Ali et al., 2014; Gujar et al., 2014; Pandit et al., 2015). To date, many homogeneous and heterogeneous catalysts have been reported for the synthesis of pyranopyrazoles under different conditions. However, some of these systems suffer from the problems of high catalyst loading, harsh conditions, and the use of toxic organic solvents. Therefore, design a green and efficient catalytic system to overcome the above limitations is a significant subject between chemists. Given the above and continuing our recent studies on the design and preparation of novel magnetic and mesoporous catalytic systems, herein novel magnetic methylene and ionic liquid-based bifunctional periodic mesoporous organosilica (BPMO) supported copper with yolk-shell structure (YS-Fe_3_O_4_@PMO/IL-Cu) was prepared and applied as an effective and recoverable catalyst for the synthesis of pyranopyrazoles under green conditions. In this BPMO, methylene functional groups are incorporated into the mesoporous walls, while ionic liquid functions are located in the mesoporous channels.

## 2 Experimental section

### 2.1 Synthesis of YS-Fe_3_O_4_@PMO NPs

For the synthesis of YS-Fe_3_O_4_@PMO, Fe_3_O_4_ NPs were first prepared according to our previous procedure (Neysi et al., 2019). Then, Fe_3_O_4_ NPs (0.25 g) were added to a reaction flask containing EtOH)16 mL (, H_2_O (36 mL), CTAB (0.72 g), pluronic P123 (17.1 g) and ammonia solution (0.9 mL, 25% wt). This was stirred at 35°C–40°C for 0.5 h. Next, tetraethyl orthosilicate (TEOS, 0.7 g) and bis(triethoxysilyl)methane (BTEM, 2.1 g) were added to the reaction flask, and stirring continued for 1 h. The resulting mixture was statically heated at 100°C for 17 h. The product was separated, washed with ethanol and water, and dried at 80°C for 7 h. Finally, to obtain a yolk-shell structure, the CTAB and pluronic P123 templates were removed by Soxhlet extraction (Zhang et al., 2008).

### 2.2 Synthesis of YS-Fe_3_O_4_@PMO/IL NPs

For this part of the study, YS-Fe_3_O_4_@PMO NPs (0.25 g) were added and ultrasonically dispersed in toluene (20 mL) at RT for 20 min. Then, 1-methyl-3-(3-trimethoxysilylpropyl) imidazolium chloride (0.15 g) was added, and the resulting mixture was refluxed under Ar atmosphere for 1 day. After cooling to room temperature, the product was collected using a magnet, washed with ethanol, dried at 75°C for 11 h, and named YS-Fe_3_O_4_@PMO/IL.

### 2.3 Synthesis of YS-Fe_3_O_4_@PMO/IL-Cu catalyst

First, the YS-Fe_3_O_4_@PMO@IL NPs (0.25 g) were sonicated in DMSO (40 mL) for 20 min. Then, Cu(OAc)_2_.4H_2_O (0.75 g) was added while stirring at RT for 1 day. The resulting mixture was then stirred at 80°C for 2 h. The product was collected using a magnet, washed with ethanol and H_2_O, dried at 75°C for 11 h, and named YS-Fe_3_O_4_@PMO/IL-Cu (Elhamifar et al., 2017). According to the ICP analysis, the loading of copper on the designed material was found to be 0.45 mmol Cu/g of YS-Fe_3_O_4_@PMO/IL-Cu.

### 2.4 Synthesis of pyranopyrazoles using YS-Fe_3_O_4_@PMO/IL-Cu catalyst

For this part of the study, YS-Fe_3_O_4_@PMO/IL-Cu catalyst (0.36 mol%) was added to a flask containing aldehyde (1 mmol), malononitrile (1 mmol), ethyl acetoacetate (1 mmol), and hydrazine hydrate (1 mmol). The reaction progress was monitored under ultrasonic conditions at RT. After the reaction was completed, the hot EtOH was added to the reaction flask, and YS-Fe_3_O_4_@PMO/IL-Cu was separated using an external magnetic field. The pure pyranopyrazoles were obtained after recrystallizing the crude mixture in EtOH.

### 2.5 IR, ^1^H and ^13^C-NMR data of pyranopyrazoles

#### 2.5.1 6-Amino-4-(2,4-dichlorophenyl)-3-methyl-1,4-dihydropyrano[2,3-c]pyrazole- 5-carbonitrile

IR (KBr, cm^-1^): 3,480 (NH), 3,253, 3,118 (NH_2_), 3,075 (=C-H stretching vibration, sp^2^), 2,927 (C-H stretching vibration, sp^3^), 2,184 (CN), 1,641 (C=N), 1,467 (C=C), 1,411 (C-O, ether), 869 (C-Cl). ^1^H-NMR (400 MHz, CDCl_3_): *δ* (ppm), 1.90 (s, 3H), 4.45 (s, 1H), 7.01 (d, 1H, j = 8 Hz), 7.11 (d, 1H, j = 8 Hz), 7.76 (s, 1H), 8.60 (s, 2H, NH_2_), 11.95 (s, 1H, NH). ^13^C-NMR (100 MHz, CDCl_3_): *δ* (ppm) 13.2, 16, 59.3, 110.0, 117.4, 126.7, 130.0, 131.0, 132.4, 135.0, 139.6, 142.6, 163.7, 177.4.

#### 2.5.2 6-Amino-4-(2-bromo-6-hydroxyphenyl)-3-methyl-1,4-dihydropyrano[2,3- c]pyrazole-5-carbonitrile

IR (KBr, cm^-1^): 3,495 (OH), 3,380 (NH), 3,255, 3,120 (NH_2_), 3,079 (=C-H stretching vibration, sp^2^), 2,917 (C-H stretching vibration, sp^3^), 2,187 (CN), 1,619 (C=N), 1,475 (C=C), 1,268 (C-O, ether), 823 (C-Br). ^1^H-NMR (400 MHz, CDCl_3_): δ (ppm) 1.94 (s, 3H), 4.53 (s,1H), 5.30 (s, 1H, OH), 6.92 (t, 1H, j = 5.8 Hz), 7.10 (d, 1H, j = 8 Hz), 7.11 (d, 1H, j = 8 Hz), 8.67 (s, 2H, NH_2_), 11.88 (s, 1H, NH). ^13^C-NMR (100 MHz, CDCl_3_): *δ* (ppm),13.3, 16.5, 59.5, 110.2, 113.4, 117.3, 123.7, 123.9, 128.7, 128.9, 139.6, 156.6, 163.9, 177.6.

## 3 Results and discussion

**SCHEME 1:**
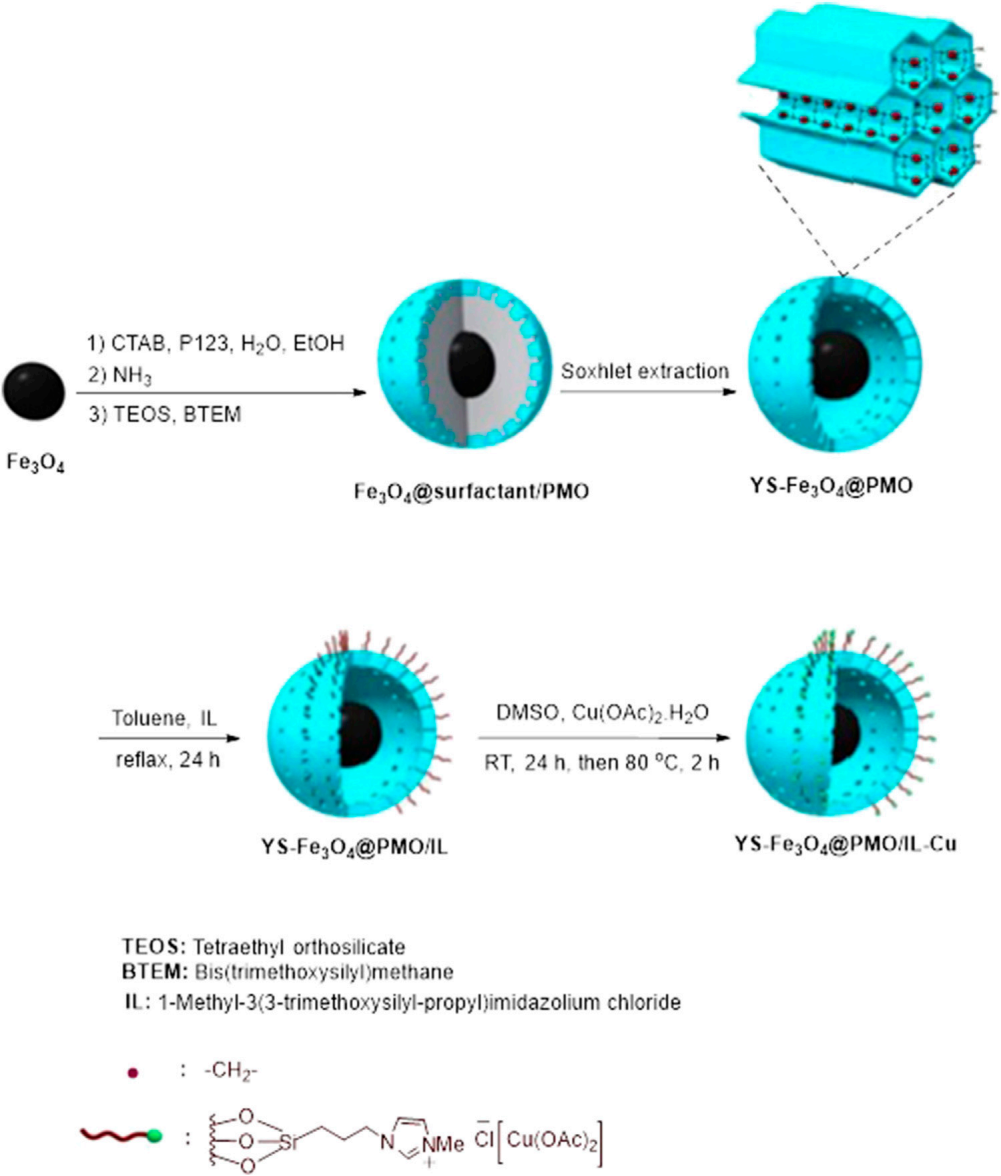
Preparation of the YS-Fe_3_O_4_@PMO/IL-Cu catalyst.

The synthesis of YS-Fe_3_O_4_@PMO/IL-Cu NPs is presented in Scheme 1. Initially, the surface of Fe_3_O_4_ NPs was coated with a periodic mesoporous organosilica shell via hydrolysis and co-condensation of TEOS and BTEM in the presence of CTAB and pluronic P123 surfactants. To obtain a yolk-shell structure, the CTAB and pluronic P123 templates were removed by Soxhlet extraction. Subsequently, the surface of YS-Fe_3_O_4_@PMO/IL NPs was modified with a complex of ionic liquid and copper salt to obtain YS-Fe_3_O_4_@PMO/IL-Cu catalyst. It is important to note that the YS-Fe_3_O_4_@PMO/IL-Cu catalyst is a multifunctional material that contains the advantages of magnetic NPs, supported ionic liquids and YS-structured mesoporous materials. For example, as shown in Scheme 1, IL moieties play a key role in the immobilization and stabilization of catalytic copper species.

**FIGURE 1:**
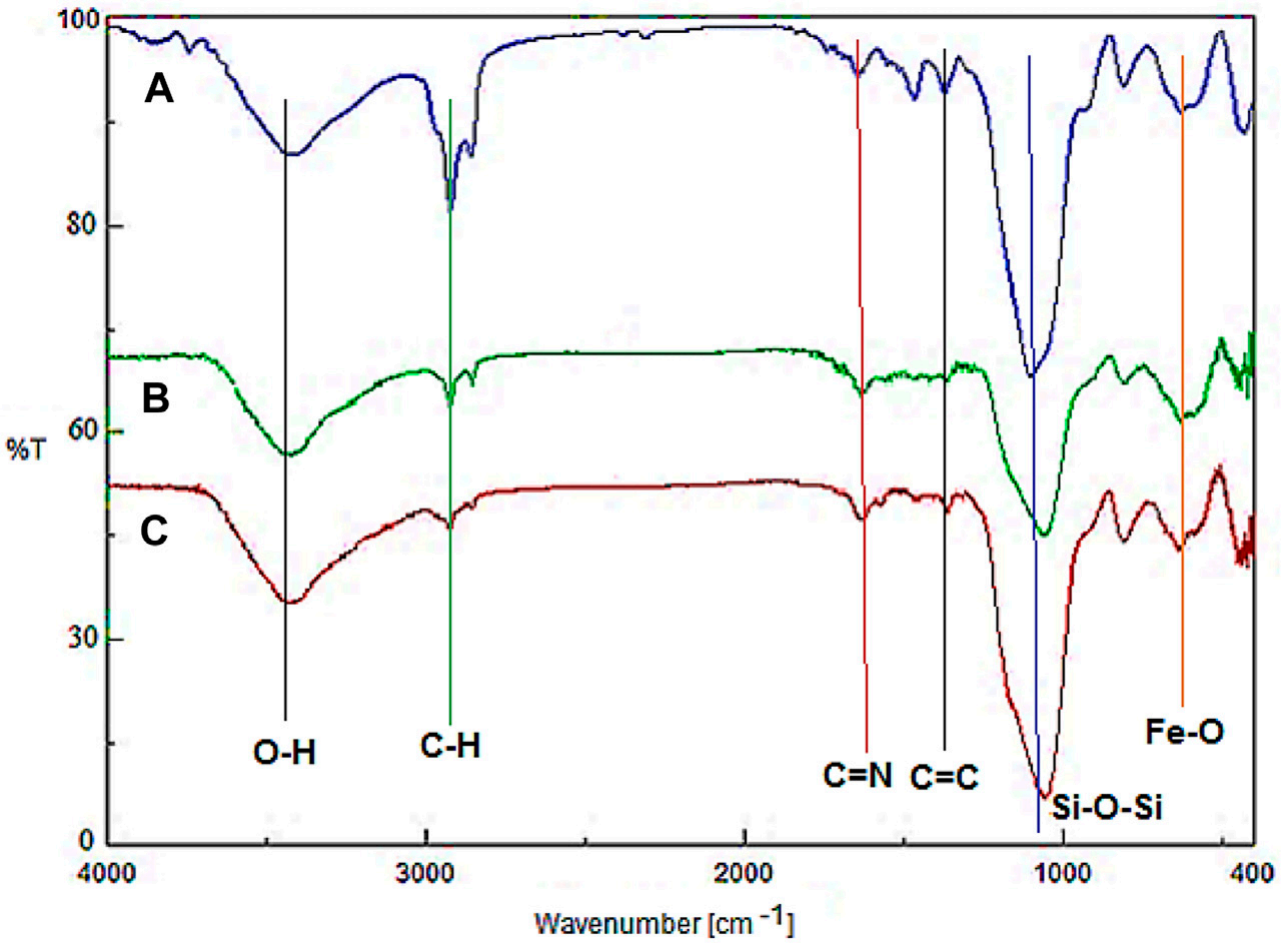
FT-IR of **(A)** Fe_3_O_4_@Surfactants@PMO, **(B)** YS-Fe_3_O_4_@PMO, and **(C)** YS-Fe_3_O_4_@PMO/IL-Cu.

Figure 1 demonstrates the FT-IR of Fe_3_O_4_@surfactants@PMO, YS-Fe_3_O_4_@PMO, and YS-Fe_3_O_4_@PMO/IL-Cu NPs. For all samples, the peaks observed at 588 and 3300–3450 cm^-1^ are related to Fe-O and O-H bonds, respectively. Also, the signals observed at 823 and 1078 cm^-1^ are assigned to the asymmetric and symmetric vibrations of the Si-O-Si bond, respectively (Figures 1A,B). It should be noted that before surfactant extraction, the sharp peaks at 2923 and 2855 cm^-1^ are due to C-H stretching vibrations of CTAB and P123 (Figure 1A). After the Soxhlet extraction, the intensity of these peaks is significantly decreased, confirming the successful elimination of surfactants (Figure 1B). In Figure 1C, the peaks at 1418 and 1625 cm^-1^ are related to C=C and C=N of imidazolium rings, respectively.

**FIGURE 2:**
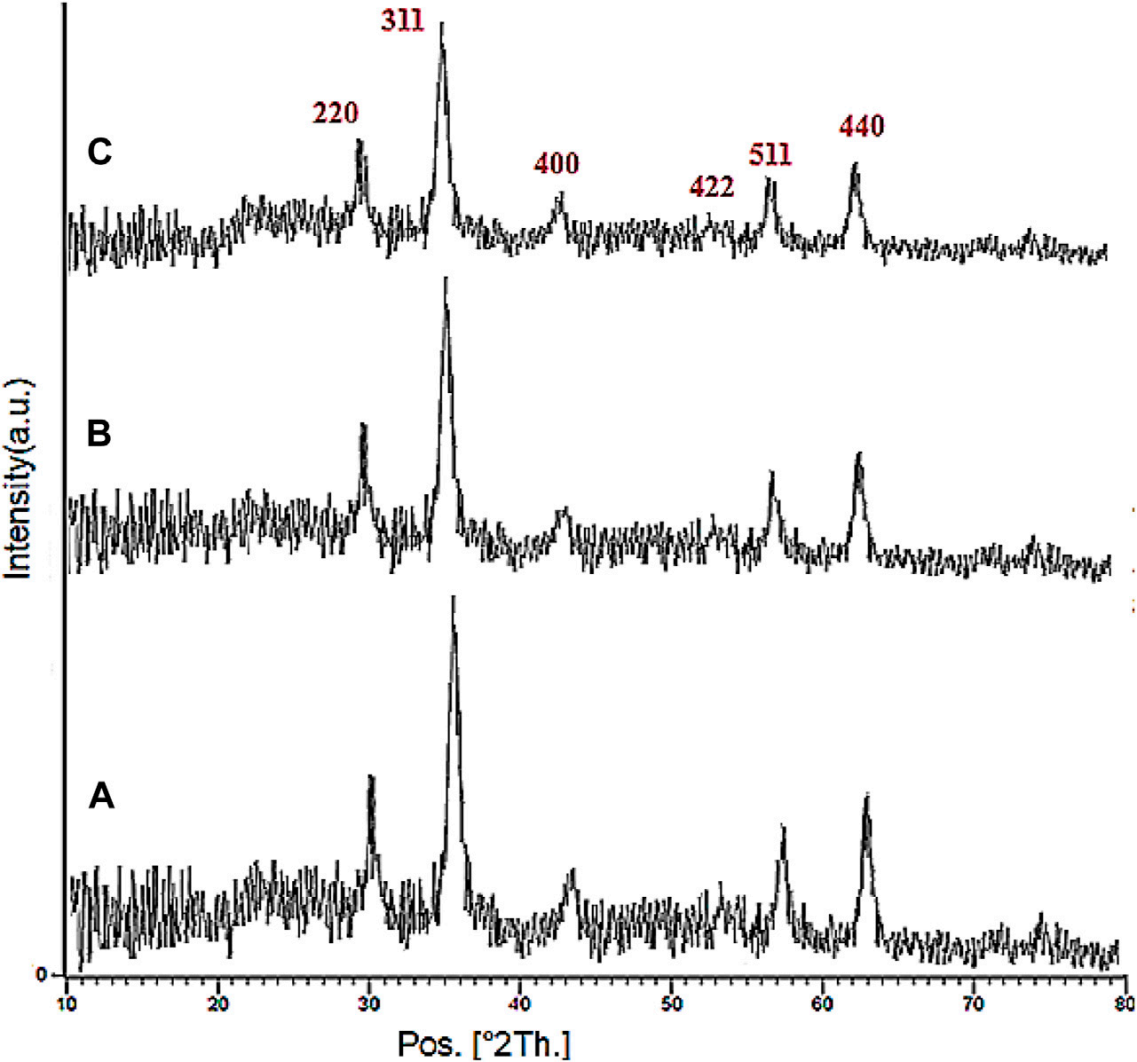
PXRD pattern of **(A)** Fe_3_O_4_, **(B)** YS-Fe_3_O_4_@PMO, and **(C)** YS-Fe_3_O_4_@PMO/IL-Cu.

The XRD analysis of the Fe_3_O_4_, YS-Fe_3_O_4_@PMO, and YS-Fe_3_O_4_@PMO/IL-Cu catalysts is displayed in Figure 2. This clearly illustrates six signals at 2Ɵ = 30.3, 35.7, 43.4, 53.8, 57.7, and 63.0°, which is in agreement with the standard XRD pattern of Fe_3_O_4_ NPs. This confirms that the Fe_3_O_4_ NPs are very stable during the preparation of the YS-Fe_3_O_4_@PMO/IL-Cu catalyst. It is also important to note that for YS-Fe_3_O_4_@PMO and YS-Fe_3_O_4_@ PMO/IL-Cu materials, the intensity of PXRD peaks is decreased, indicating the successful modification of magnetite NPs with Me-PMO, IL, and copper moieties. (Figure 2).

**FIGURE 3:**
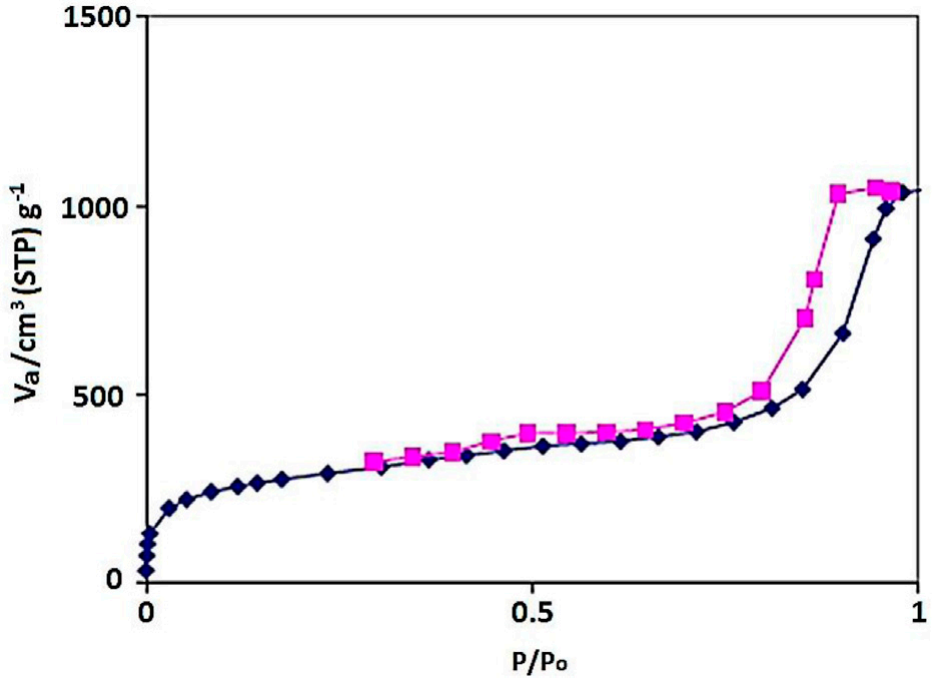
N_2_ adsorption–desorption isotherm of YS-Fe_3_O_4_@PMO/IL-Cu.

The N_2_ adsorption–desorption isotherm of the YS-Fe_3_O_4_@PMO/IL-Cu showed a type IV isotherm with an H1 hysteresis loop, which is characteristic of ordered mesostructures with high regularity (Figure 3). Also, the BET surface area, average pore size, and total pore volume of the designed YS-Fe_3_O_4_@PMO/IL-Cu nanocomposite were found to be 659 m^2^/g, 7.6 nm, and 1.30 cm^3^/g, respectively. These results demonstrate the good formation of an ordered PMO shell for YS-Fe_3_O_4_@PMO/IL-Cu.

**FIGURE 4:**
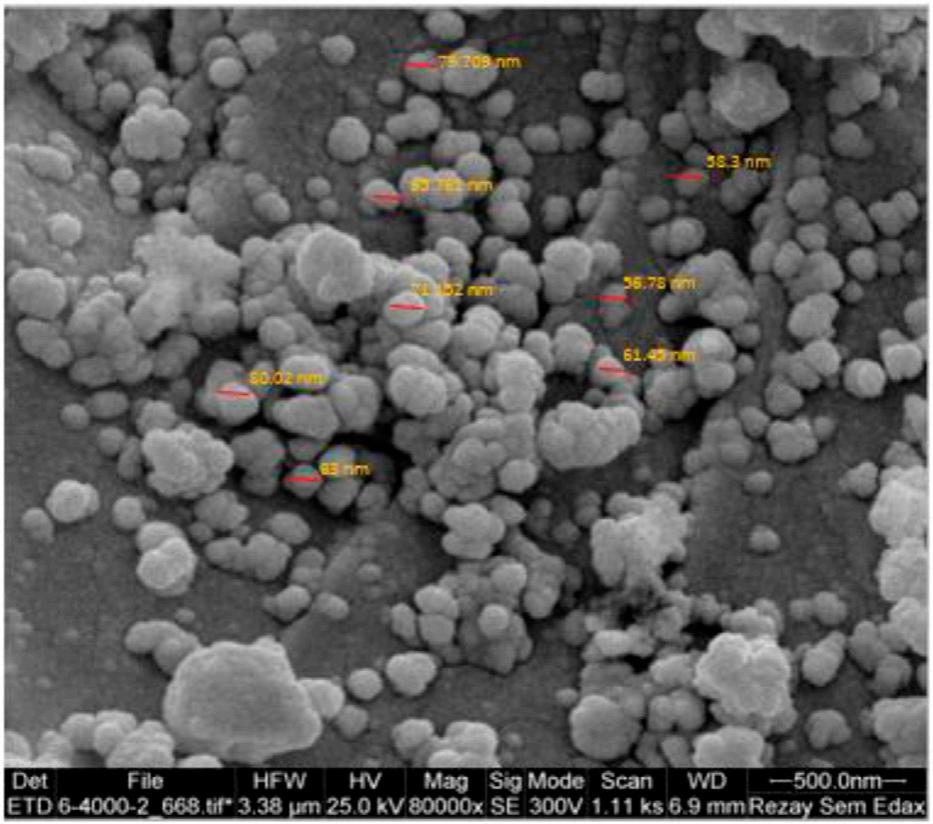
SEM analysis of the YS-Fe_3_O_4_@PMO/IL-Cu catalyst.

The SEM image of the YS-Fe_3_O_4_@PMO/IL-Cu catalyst showed the presence of uniform particles with spherical structure and an average size of 70 nm (Figure 4). These are very good NPs for catalytic and adsorption processes.

**FIGURE 5:**
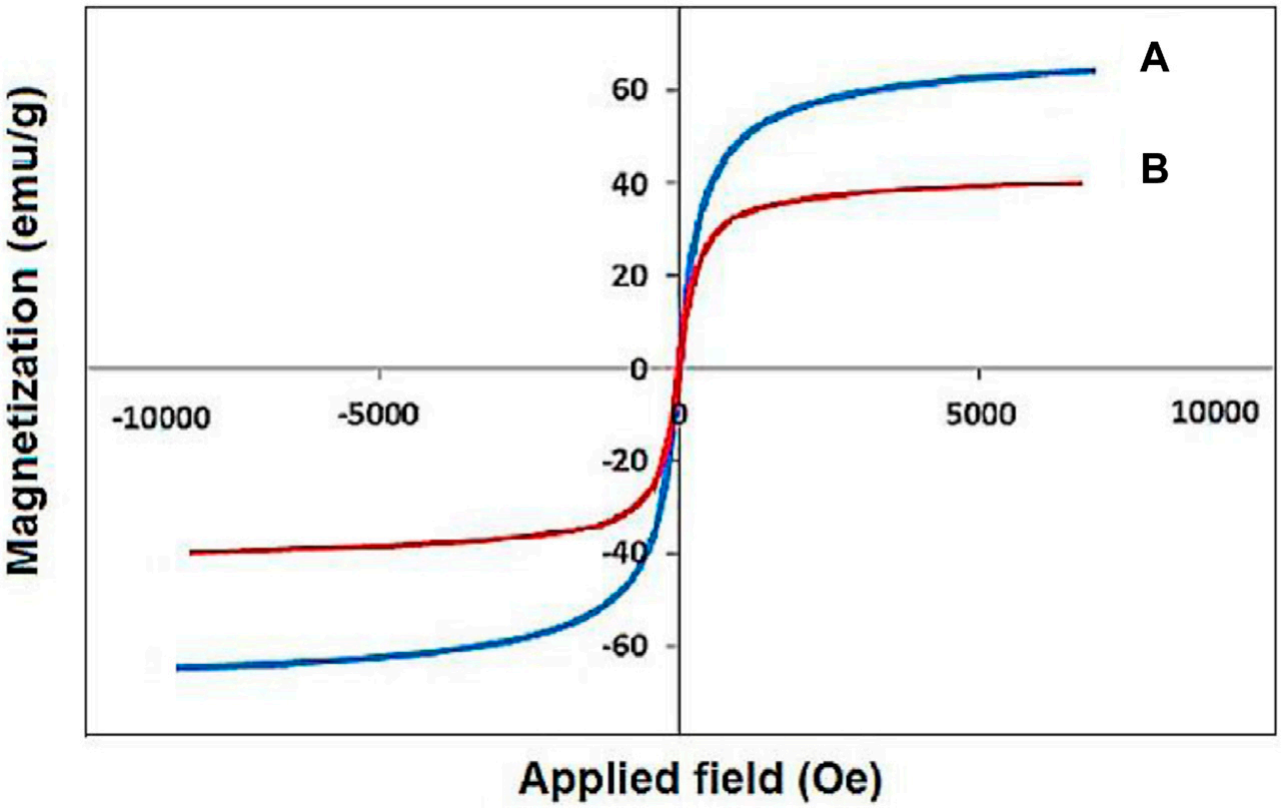
The VSM analysis of (A) Fe_3_O_4_ and (B) YS-Fe_3_O_4_@PMO/IL-Cu.

The VSM analysis showed a saturation magnetization of about 30 emu·g^−1^ for the designed YS-Fe_3_O_4_@PMO/IL-Cu nanocatalyst, lower than that of pure magnetic iron oxide NPs (Figure 5) (Norouzi et al., 2020). This proves the successful coating of PMO shell on magnetite NPs and also confirms the high magnetic properties of the catalyst, which is an excellent characteristic in the catalytic field.

**FIGURE 6:**
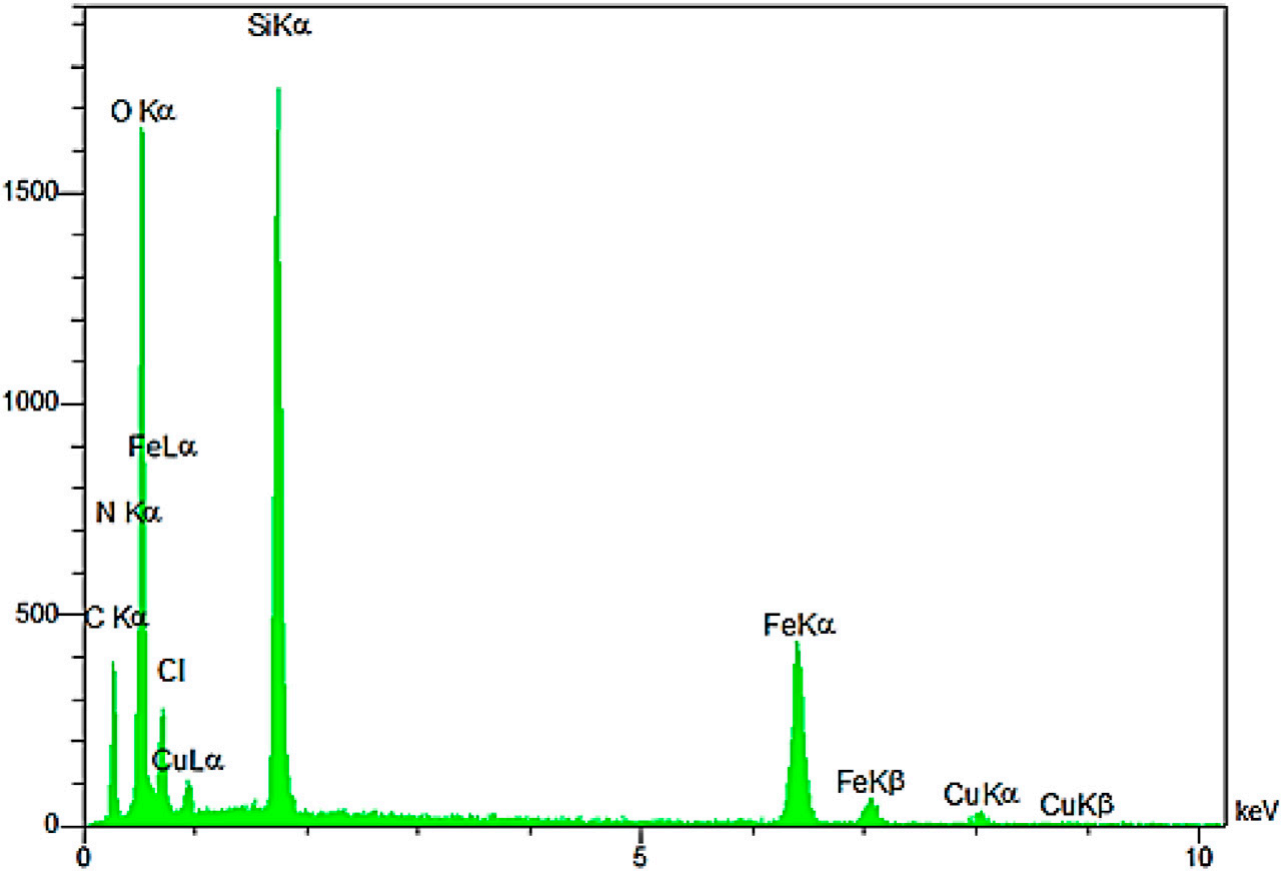
The EDX analysis of the YS-Fe_3_O_4_@PMO/IL-Cu catalyst.

The EDX pattern confirmed the presence of the desired elements in the YS-Fe_3_O_4_@PMO/IL-Cu catalyst (Figure 6). This analysis illustrated the signals of C, Si, N, Cu, Fe, and O elements in the catalyst, proving the successful incorporation and immobilization of the expected inorganic and organic moieties into/onto Fe_3_O_4_ NPs.

**FIGURE 7:**
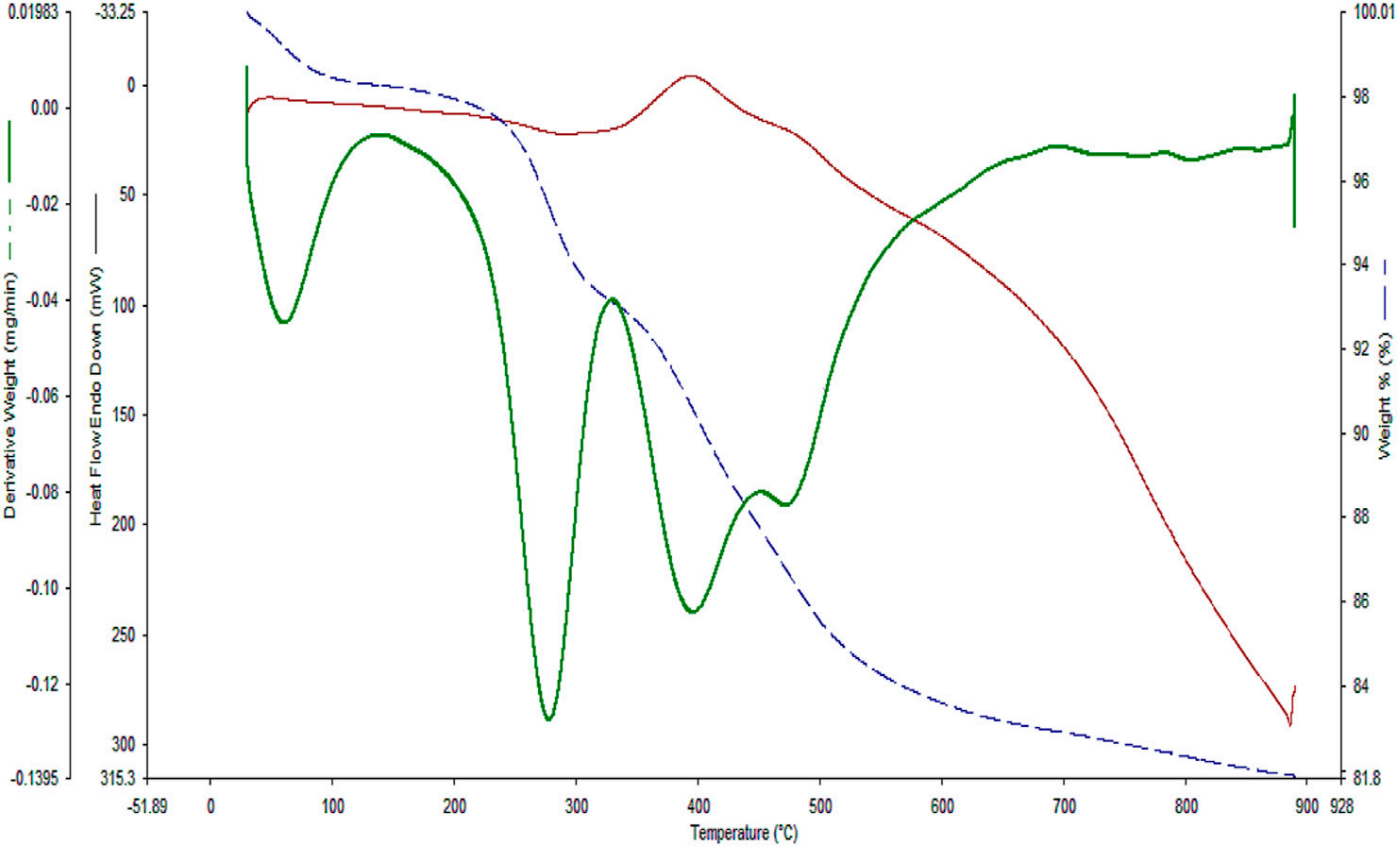
The TG analysis of the YS-Fe_3_O_4_@PMO/IL-Cu catalyst.

In the next step, TGA analysis was conducted to evaluate the thermal stability of the YS-Fe_3_O_4_@PMO/IL-Cu catalyst (Figure 7). The first weight loss below 120°C is due to the loss of water and alcoholic solvents left over from the synthesis process. Another weight loss at 200°C–320°C is related to the decomposition of the remaining CTAB and P123 surfactants. The highest weight loss, observed at 325°C–650°C, is attributed to the removal of methylene and ionic liquid functional groups, which are incorporated/immobilized in/on the structure of YS-Fe_3_O_4_@PMO/IL-Cu nanocomposite.

After characterizing the YS-Fe_3_O_4_@PMO/IL-Cu catalyst, its application in the synthesis of dihydropyrano [2, 3-*c*]pyrazoles was investigated. For this part of the study, the condensation between malononitrile, PhCHO, ethyl acetoacetate, and hydrazine hydrate was selected as a model reaction. The effects of the solvent and catalyst loading were studied at RT under ultrasonic conditions. As displayed in Table 1, the effects of different solvents such as EtOH, CH_3_CN, *n*-Hexane, DMF, H_2_O, and solvent-free media were studied, and the best results were obtained in H_2_O at 25°C (Table 1, entries 1–6). The effect of catalyst loading was also investigated, with the best yield obtained in the presence of 0.36 mol% of YS-Fe_3_O_4_@PMO/IL-Cu. According to these results, the use of 0.36 mol% of YS-Fe_3_O_4_@PMO/IL-Cu in H_2_O at 25°C under ultrasonic irradiation was chosen as the optimum condition. In order to prove whether the cupper centers act as catalytic sites or not, in the next study the reaction was carried out using Cu-free Fe_3_O_4_, YS-Fe_3_O_4_@PMO, and YS-Fe_3_O_4_@PMO/IL materials under the same conditions as YS-Fe_3_O_4_@PMO/IL-Cu (Table 1, entries 10–12). The result showed that for all Cu-free samples, only a low yield of the desired product was obtained, indicating that the reaction is mainly catalyzed by immobilized copper sites.

**TABLE 1:** Effect of solvent and catalyst loading in the synthesis of dihydropyrano[2, 3-*c*]pyrazole^a^.

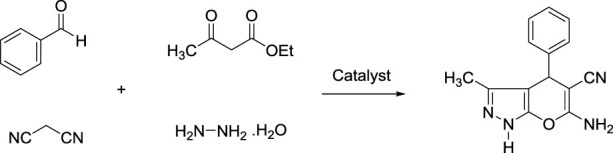			
Entry	Solvent	Catalyst (mol%)	Yield (%)
1	—	YS-Fe_3_O_4_@PMO/IL-Cu (0.36)	28
2	EtOH	YS-Fe_3_O_4_@PMO/IL-Cu (0.36)	65
3	CH_3_CN	YS-Fe_3_O_4_@PMO/IL-Cu (0.36)	14
4	DMF	YS-Fe_3_O_4_@PMO/IL-Cu (0.36)	50
5	*n*-Hexane	YS-Fe_3_O_4_@PMO/IL-Cu (0.36)	<10
6^b^	H_2_O	YS-Fe_3_O_4_@PMO/IL-Cu (0.36)	95
7	H_2_O	YS-Fe_3_O_4_@PMO/IL-Cu (0.45)	95
8	H_2_O	YS-Fe_3_O_4_@PMO/IL-Cu (0.18)	68
9	H_2_O	YS-Fe_3_O_4_@PMO/IL-Cu (0.09)	35
10	H_2_O	YS-Fe_3_O_4_@PMO/IL (0.008 g)	23
11	H_2_O	YS-Fe_3_O_4_@PMO (0.008 g)	21
12	H_2_O	Fe_3_O_4_ (0.008 g)	35

^a^ All reactions were performed at RT, for 10 min.

^b^ Optimum conditions.

After optimizing the different parameters, the efficiency of the YS-Fe_3_O_4_@PMO/IL-Cu nanocatalyst was evaluated by using different aldehyde substrates for the preparation of pyrazole derivatives. As seen in Table 2, all investigated aldehydes were converted to their corresponding products in high yields. These results confirm the high efficiency of YS-Fe_3_O_4_@PMO/IL-Cu for the preparation of a wide range of biologically active pyranopyrazoles.

**TABLE 2:** Synthesis of pyranopyrazoles by using YS-Fe_3_O_4_@PMO/IL-Cu^a^.

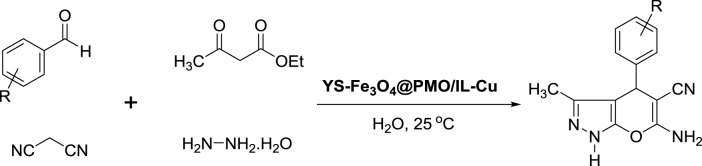					
Entry	Aldehyde	Time (min)	Yield (%)^b^	Found M. P.	Reported M. P.
1	C_6_H_5_CHO	10	95	241–243	240–243^33^
2	4-MeO-C_6_H_4_CHO	15	89	206–208	206–209^33^
3	4-Me-C_6_H_4_CHO	20	89	174–176	176–177^28^
4	3-HO-C_6_H_4_CHO	17	90	262–264	260–262^33^
5	4-Br-C_6_H_4_CHO	12	89	182–184	180–182^33^
6	4-CN-C_6_H_4_CHO	10	96	197–199	196–198^30^
7	4-NO_2_-C_6_H_4_CHO	10	87	191–193	194–196^28^
8	4-Cl-C_6_H_4_CHO	8	93	231–233	233–235^32^
9	2,4-diCl-C_6_H_3_CHO	25	85	217–219	New
10	2-Br-6-HO-C_6_H_3_CHO	50	86	271–273	New

^a^ Conditions: ethyl acetoacetate (1 mmol), malononitrile (1 mmol), benzaldehyde (1 mmol), hydrazine hydrate (1 mmol), and catalyst (0.36 mol%) in H_2_O (8 mL) at 25°C.

^b^ Isolated yields.

**FIGURE 8:**
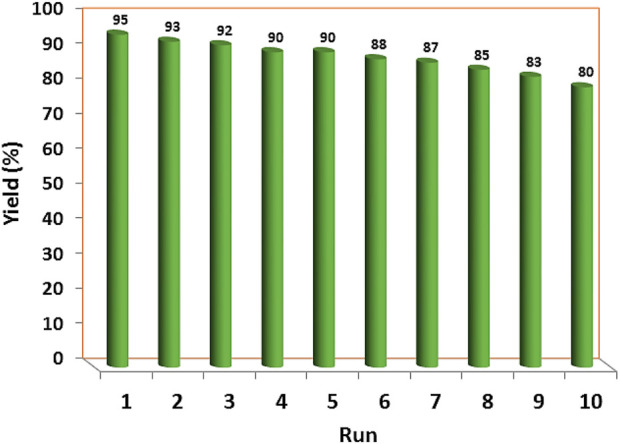
The recoverability and reusability of YS-Fe_3_O_4_@PMO/IL-Cu.

The recoverability and reusability of the YS-Fe_3_O_4_@PMO/IL-Cu catalyst were investigated in the condensation of malononitrile, ethyl acetoacetate, benzaldehyde, and hydrazine hydrate under optimized conditions. For this purpose, at the end of the reaction, the catalyst was magnetically removed, washed, and reused in the next run under the same conditions as in the first step. The results indicated that the synthesized catalyst can be recovered and reused at least 9 times without significant loss of efficiency (Figure 8).

A leaching test was then performed to investigate the nature of the catalyst under the reaction conditions. For this purpose, the model reaction was selected as the test. After about 50% of the process was completed, the catalyst was removed using an external magnet, and the reaction progress of the residue was monitored for 60 min. The result demonstrated no further progress of the reaction, confirming no leaching of the active catalytic species and also the heterogeneous nature of the designed catalyst. This result confirms the successful immobilization of the copper moieties on the material framework.

Next, a comparative study was performed between the activity of the YS-Fe_3_O_4_@PMO/IL-Cu catalyst and several identified catalysts in the synthesis of pyranopyrazoles (Table 3). The results showed that our designed catalyst is better than other catalysts in terms of catalyst loading, reaction time, and recovery numbers. These findings are attributed to the magnetic nature, mesoporous structure, supported ionic liquids, and high stability of the designed YS-Fe_3_O_4_@PMO/IL-Cu nanocatalyst.

**TABLE 3:** Comparative study between the activity of the YS-Fe_3_O_4_@PMO/IL-Cu catalyst and several identified catalysts in the synthesis of pyranopyrazoles.

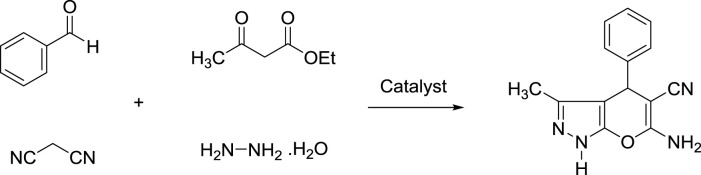				
Entry	Catalyst	Conditions	Recovery numbers	Ref
1	L-proline	H_2_O, cat. (10 mol%), reflux, 10 min	-	Mecadon et al. (2011)
2	SiO_2_-TMG	Neat, cat. (10 mol%), 100°C, 30 min	4	Atar et al. (2014)
3	Fe_3_O_4_@SiO_2_-HMTA-SO_3_H	Solvent free, cat. (0.03 g), RT, 12 min	4	Ghorbani-Vaghei and Izadkhah (2018)
4	Fe_3_O_4_@SiO_2_-EP-NH-HPA	H_2_O, cat. (0.02 g), RT, 5 min	7	Hosseini Mohtasham and Gholizadeh (2020)
5	Fe_3_O_4_	H_2_O, cat. (0.015 g), RT, 60 min	8	Ali et al. (2014)
6	YS-Fe_3_O_4_@PMO/IL-Cu	H_2_O, cat. (0.36 mol%), RT, 10 min	9	This work

## 4 Conclusion

In this study, the magnetic YS-Fe_3_O_4_@PMO/IL-Cu catalyst was prepared and identified by using PXRD, FT-IR, TGA, EDX, ICP, SEM, nitrogen sorption, and VSM analyses. The TGA, EDX, and FT-IR analyses demonstrated the high chemical and thermal stability of YS-Fe_3_O_4_@PMO/IL-Cu. The VSM and PXRD analyses showed very good magnetic properties of the material. The nano dimensions and particle size of this catalyst were confirmed using SEM analysis. The nitrogen-sorption diagram also showed a mesoporous structure for the designed catalyst. The YS-Fe_3_O_4_@PMO/IL-Cu nanocomposite was used as a powerful catalyst in the synthesis of biologically active pyranopyrazoles, giving the desired products in high yields and selectivity. Moreover, the YS-Fe_3_O_4_@PMO/IL-Cu catalyst was easily recovered and reused at least 9 times without any significant decrease in its efficiency.

## Data Availability

The original contributions presented in the study are included in the article/supplementary material, further inquiries can be directed to the corresponding author.
